# The Effect of Tobacco Smoking and Smoking Cessation on Urinal miRNAs in a Pilot Study

**DOI:** 10.3390/life10090191

**Published:** 2020-09-10

**Authors:** Zdenka Navratilova, Stanislav Losse, Pavla Petrova, Katerina Sikorova, Alzbeta Chabronova, Martin Petrek

**Affiliations:** 1 Department of Pathological Physiology, Faculty of Medicine and Dentistry, Palacky University, Hnevotinska 3, 775 15 Olomouc, Czech Republic; Katerina.Sikorova@fnol.cz (K.S.); chabronova.alzbeta@gmail.com (A.C.); 2 Department of Respiratory Medicine, University Hospital, 779 00 Olomouc, Czech Republic; stanislav.losse@fnol.cz; 3 Department of Clinical Biochemistry, University Hospital, 779 00 Olomouc, Czech Republic; Pavla.Petrova@fnol.cz; 4 Laboratory of Cardiogenomics (LEM), University Hospital, 779 00 Olomouc, Czech Republic; 5 Institute of Molecular and Translational Medicine, Faculty of Medicine and Dentistry, Palacky University, Hnevotinska 3, 775 15 Olomouc, Czech Republic

**Keywords:** urine, microRNAs, nicotine, creatinine, quitting of smoking

## Abstract

The diseases associated with tobacco smoking affect miRNAs and small single-stranded non-coding RNAs. However, there are no data on urinal miRNAs in healthy smokers. We searched for the possible effect of smoking and smoking cessation on miRNA urine expression. For screening, Affymetrix miRNA 4.0 arrays were used in 33 urine samples obtained from six never smokers and from current smokers in three time-points before smoking cessation (*n* = 10), after short time abstinence (3–8 weeks), and after long-term abstinence (1 year). For validation, a quantitative (q) polymerase chain reaction (PCR) method was used in 93 urine samples obtained from 18 never smokers and 25 current smokers in three time-points before smoking cessation, after short time abstinence (3–8 weeks), and after long-term abstinence (1 year). In screening analysis, 5 miRNAs (hsa-miR-3620-5p, hsa-miR-3613-5p, hsa-miR-3921, hsa-miR-5094, and hsa-miR-337-3p) were dysregulated in current vs. never smokers after multiple testing corrections. Smoking cessation was accompanied by miRNA dysregulation that did not reach a significant level after a multiple testing correction. In validation analysis, three miRNAs correlated with cotinine, but they were affected neither after smoking cessation nor between current and never smokers. Our whole-genome screening of 2.578 miRNAs and validation suggest that tobacco smoking has no or only a small effect on urinal miRNAs.

## 1. Introduction

Tobacco smoking is a primary cause of COPD (chronic obstructive pulmonary disease) in developed countries and deteriorates the course of other respiratory diseases, e.g., asthma [1,2]. Lung cancer is also more often among tobacco smokers [3,4]. Besides the respiratory diseases, tobacco smoking is a risk factor for several diseases affecting cardiovascular, gastrointestinal, urogenital, and other organ systems [5,6]. The pathophysiological mechanism of how tobacco smoking contributes to these serious diseases is the subject of the current intensive investigation that involves the dysregulation of miRNAs [7,8].

miRNAs are small single-stranded (20–24 nucleotides long) non-coding RNAs that bind to complementary sequences within targeted mRNAs in the RISC *(RNA-induced silencing complex)* complex and, thus, inhibit the specific translation process [9,10]. Besides the intracellular role, the extracellular miRNAs can provide cell-cell communication. However, some extracellular miRNAs may simply be a waste product without any biological properties [10]. Both intracellular and extracellular miRNAs have been considered as possible biomarkers of various diseases associated with smoking.

In particular, several studies investigated the expression profile of miRNAs associated with COPD [11,12], asthma [13,14], and lung carcinoma [15,16,17]. Regarding a biological function, for instance, the smoking-induced overexpression of miR-216b was shown to increase non-small cell lung cancer cell growth by downregulating Smad3 and inhibiting transforming growth factor beta-induced tumor suppressor function, and also to induce resistance to platinum-based therapy [18]. The alterations of miRNAs have also been associated with several extra-pulmonary diseases, e.g., chronic kidney injury [19,20,21,22], renal cell, and bladder carcinoma [23,24,25,26,27,28,29,30,31] common among smokers. The extra-pulmonary alternations of miRNAs indicate new biomarkers and also one of the possible mechanisms of how tobacco smoking increases the risk for developing these diseases. However, there is no information on whether tobacco smoking has any physiological effect on urinal miRNAs that had been reported to be a relevant source of non-invasive biomarkers in the smoking-associated disease. We hypothesize that some urine miRNAs can be affected by tobacco smoking and considered as biomarkers of the smoking status.

## 2. Materials and Methods

### 2.1. Study Population

Urine samples were obtained from 43 healthy volunteers recruited at Nicotine dependence Center, Department of Respiratory Medicine and TBC, University Hospital in Olomouc, the Czech Republic. Of them, 18 subjects were never smokers and 25 subjects were current smokers who quit successfully. To study the effect of short-term and long-term abstinence from tobacco smoking, urine samples were collected repeatedly in three time points before smoking cessation (*n* = 25), 3–8 weeks (min.-max., mean = 5 weeks) after smoking cessation (*n* = 25), and one year after smoking cessation (*n* = 25). A genome-wide screening study was performed in six never smokers and 10 current smokers. Of them, 10 subjects quit successfully and provided their samples after a short-term of abstinence (3–8 weeks, mean = 5 weeks). Regarding long-term abstinence (1 year), three ex-smokers failed and seven ex-smokers were only included for the screening.

Smoking status was confirmed by the assessment of urine cotinine in each sample and by a breath CO test before each sample collection (Breath CO monitor for Smoking Cessation BMC 2000). Smoking history and demographic characteristics were obtained using self-administered questionnaires and the Fagerstrom Test for Nicotine Dependence. Counseling and medication supported smoking cessation. Based on comorbidities, contra-indications, and patients’ preferences, varenicline (an active ingredient of Champix) and/or bupropion (an active ingredient of Wellbutirm) were administrated in 76% and/or 32% of the current smokers in combination with nicotine spray (76%), gum (36%), and patch (8%). The schedule dosage for varenicline was 0.5 mg per day for three days. Then the dosage was 0.5 mg twice a day for four days. Lastly, the dosage was increased up to 1 mg twice per day [32]. A starting dose of bupropion was 150 mg taken once a day for three days and then increased to 150 mg taken twice a day. The most frequent comorbidity was arterial hypertension (28%), which was followed by gastroesophageal reflux (20%), asthma (16%), diabetes mellitus (16%), chronic obstructive pulmonary disease (8%), and others, e.g., autoimmune thyroiditis, ulcerative colitis, spondylitis, obesity, psoriasis, sinusitis, headache, and depression. Exclusion criteria included an acute disease or a chronic disease of the urinary tract. Informed consent for the anonymous usage of the samples for the purposes of the study was signed by all enrolled subjects. The study was performed with the approval of the Ethical committees of Medical Faculty PU and University Hospital, Olomouc.

### 2.2. Sample Collection and Proceeding

Midstream urine samples were obtained during the midday and processed within 1 h. To avoid contaminating the samples with miRNAs from kidney and bladder cells, the urine samples were centrifuged at 3000 *g* for 10 min at room temperature, aliquoted, and stored at −80 °C. Spectrophotometry was used to assess creatinine levels on the Cobas 8000 module c702 Analyzer (Hitachi-Roche). Urine cotinine was assessed by using the Immunolite Nicotine Metabolite kit on an IMMULITE 2000 Analyzer (Siemens Healthineers, Erlangen, Germany) by following the protocol of the manufacturer. The RNA concentrations and quality were measured by NanoDrop 2000 Spectrophotometer (Thermo Fisher Scientific, Waltham, MA, USA). The RNA integrity number (RIN) was assessed with the Agilent RNA 6000 Pico Kit using the Agilent 2100 Bioanalyzer System (Agilent, Santa Clara, CA, USA).

### 2.3. miRNA Microarray Processing and qPCR

For the genome-wide miRNA expression profiling, small RNAs (smaller than 1000 nt) were purified by using the miRCURY™ RNA Isolation Kit for Biofluids (Exiqon, Aarhus, Denmark), according to the manufacturer’s protocol. After ethanol precipitation, 130 ng of the small RNA was used in all tested samples (*n* = 33). Experiments were performed with GeneChipTM miRNA 4.0 arrays by following the manufacturer’s instructions (Affymetrix, Santa Clara, CA, USA). Microarrays were scanned with GeneChip Scanner 3000 7G (Affymetrix, Santa Clara, CA, USA).

For the qPCR, total miRNA was isolated using the Urine microRNA Purification Kit (Norgen Biotek Corp., Thorold, ON, Canada), according to the manufacturer’s protocol. Additionally, 10 ng of total RNA was used as an input per one qPCR in all tested samples (*n* = 93). Using a TaqMan miRNA reverse transcription kit (Applied Biosystems, Foster City, CA, USA), cDNA was synthesized based on the single specific primer approach. To pre-amplify miRNAs, TaqMan PreAmp Master Mix was used with the specific primers from the 20x TaqMan MicroRNA Assay (Applied Biosystems, Foster City, CA, USA). 20× TaqMan MicroRNA Assays and TaqMan 2× Universal PCR Master Mix (Applied Biosystems, Foster City, CA, USA) were used to perform qPCR of individual miRNAs (LightCycler^®^ 480 Instrument, Roche, Basel, Switzerland). The relative expression data were determined by normalizing four miRNAs (hsa-miR-3613-5p, hsa-miR-3921, hsa-miR-5094, hsa-miR-337-3p) to endogenously control RNU6B.

### 2.4. Data Analysis and Statistics

The Affymetrix raw data (.cel files) were normalized using the robust multichip average algorithm from “oligo” Bioconductor package [33] in R [34]. Normalized expression values were further analyzed by the “limma” package [35] approach to identify differentially expressed miRNAs between different groups of patients by applying linear models for microarrays (limma) methodology and the moderated t-test. Obtained P values of group comparisons were adjusted for multiple testing using the Benjamini–Hochberg correction (*p* < 0.05). No filter on a minimal level of urinary miRNAs was applied in the search of significantly changed miRNAs. The particular miRNA expression is provided in Table S1. Urine creatinine levels were given as an additional parameter in the linear model to adjust miRNA expression levels.

The Wilcoxon matched-pairs signed-rank test and the Mann-Whitney test (GraphPad Prism) compared the quantitative PCR miRNA data, cotinine, and exhaled CO levels between the studied groups (GraphPad Prism).

## 3. Results

### 3.1. Urine Cotinine

**Figure 1:**
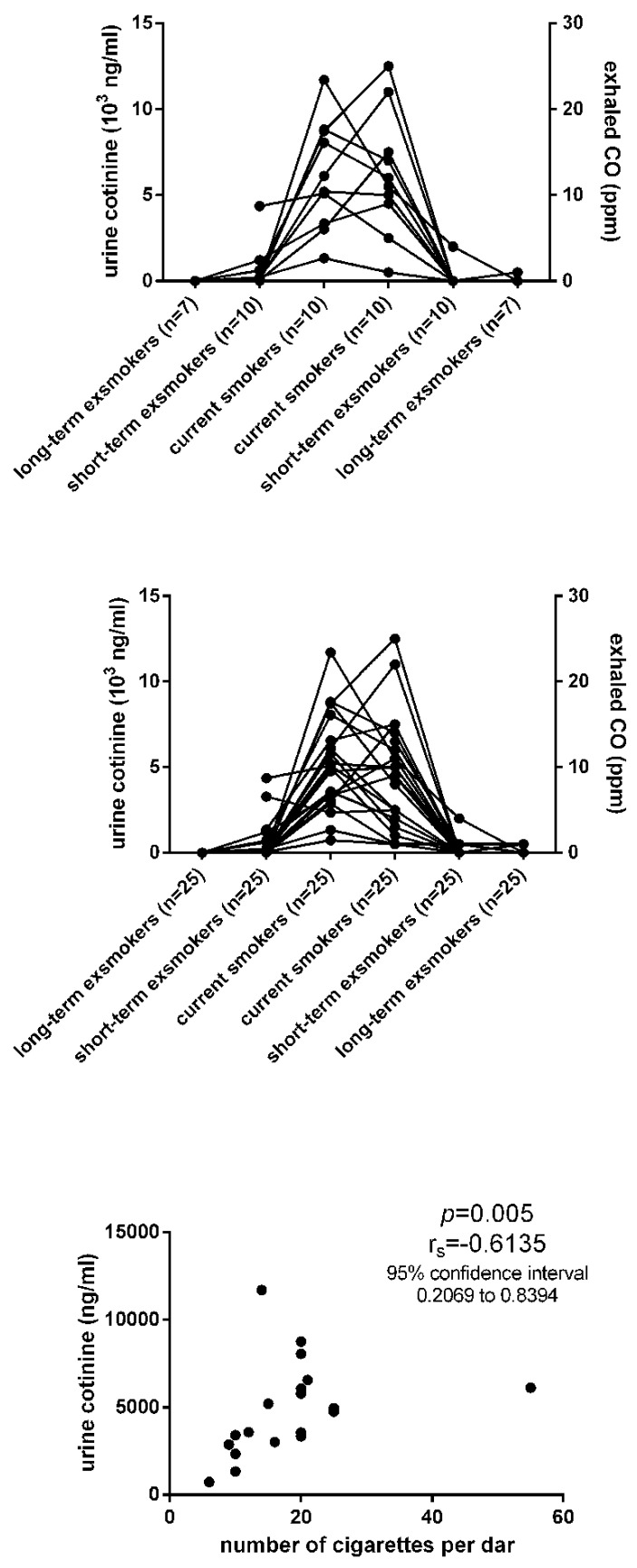
The comparison of urine cotinine levels and exhaled CO (carbon monoxide, ppm, parts per million) among current smokers and ex-smokers in a screening analysis and validation analysis. The correlation between the number of CPD (cigarettes per day) and urine cotinine level in a validation analysis.

For screening analysis, the current smokers had the urine cotinine in the range of 1325 to 11,712 ng/mL (mean, SD, 6140, 3184) that decreased to 10–4359 ng/mL (684, 1346) in short-term ex-smokers and to 10–54 ng/mL (20, 16) in long-term ex-smokers (Figure 1, Table 1). Furthermore, the current smokers had exhaled CO (carbon monoxide) in the range of 1 to 25 ppm (parts per million) (mean, SD; 12.4, 7.183) that decreased to 0–4 ppm (0.400, 1.265) in short-term ex-smokers and 0–1 ppm (0.286, 0.488) in long-term ex-smokers (Figure 1, Table 1). For validation analysis, the current smokers had the urine cotinine in the range of 736 to 11,712 ng/mL (mean, SD; 4851, 2689) that decreased to 12–3285 ng/mL (546, 875) in short-term ex-smokers and 14–54 ng/mL (24, 15) in long-term ex-smokers (Figure 1, Table 2). They had exhaled CO in the range of 1 to 25 ppm (mean, SD; 8.857, 6.139), which decreased to 0–4 ppm (0.286, 0.810) in short-term ex-smokers and 0–1 ppm (0.107, 0.315) in long-term ex-smokers (Figure 1, Table 2). All subjects referred to as the never smokers in our study had their urine cotinine below 10 ng/mL (Table 1 and Table 2). The never smokers had a lower level of urine cotinine than current smokers (*p* = 0.0002 and *p* = 0.0001), but similar levels in comparison with both groups of the short-term and long-term ex-smokers. The current smokers had higher urine cotinine levels than both groups of the short-term (*p* = 0.0001) and long-term ex-smokers (*p* = 0.0001). Furthermore, the current smokers had higher exhaled CO than both groups of the short-term (*p* = 0.0001) and long-term ex-smokers (*p* = 0.0003). Neither urine cotinine nor exhaled CO differed between the short-term and long-term ex-smokers (*p* > 0.05). The urine cotinine correlated with the number of cigarettes per day (*p* = 0.005, Figure 1). We did not observe any correlation between the urine cotinine and the total score of the Fagerstrom Test for Nicotine Dependence (*p* = 0.07).

### 3.2. Urine miRNA Pattern

**Figure 2:**
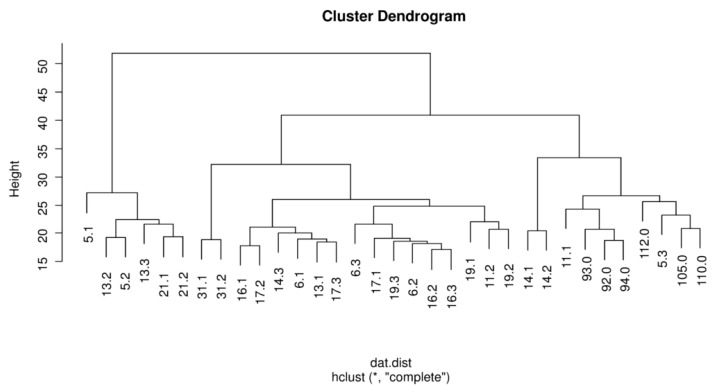
Unsupervised clustering of 33 smokers. Legend: The number following the decimal point refers to smoking status as following: 0, never smoker, 1, current smokers, 2, short term ex-smokers, and 3, long-term ex-smokers.

Unsupervised cluster analysis was used to visualize the patterns on the expression of the 2578 mature miRNA in 33 subjects (Figure 2). Figures S1–S5 show the distinct levels of miRNAs with abundant miRNA in red, miRNA detected at a moderate level in black, and miRNA present in very low quantities in green.

### 3.3. The Urine miRNA Profiles of Current/Ex-Smokers Versus Never Smokers in a Baseline Design Study

Using the criteria of *p* < 0.005, 37 miRNAs were dysregulated in current smokers compared to never smokers (Figure S1). A total of 28 miRNAs were dysregulated in the subjects who experienced short-term abstinence vs. never smokers (Figure S2). Nineteen miRNAs were dysregulated in the subjects who experienced long-term abstinence vs. never smokers (Figure S3).

After multiple testing corrections (*pc* < 0.05), 5 miRNAs were dysregulated (−0.5849 > log fold-change > +0.5849) in current smokers vs. never smokers (Table 2). Except for one up-regulated miRNAs (hsa-miR-3620-5p), all miRNAs were decreased in current smokers (hsa-miR-3613-5p, hsa-miR-3921, hsa-miR-5094, and hsa-miR-337-3p) (Table 2). The same miRNAs were also decreased in the ex-smokers who experienced short-term abstinence vs. never smokers (Table 2). In addition, hsa-miR-1298-3p was decreased in the ex-smokers who experienced short-term abstinence vs. never smokers (Table 2). Only hsa-miR-3613-5p was decreased in the ex-smokers who experienced long-term abstinence vs. never smokers (Table 2).

### 3.4. The Effect of Short-Term and Long-Term Abstinence in the Same Patient

Using the criteria of *p* < 0.005, the smoking cessation followed by the short-term abstinence (3–8 weeks) affected 18 mature miRNAs (Figure S4). The smoking cessation followed by the long-term abstinence (1 year) affected 11 mature miRNAs (Figure S5). Neither short-term nor long-term abstinence showed a significant effect on urinal miRNAs after multiple testing corrections (*pc* < 0.05).

### 3.5. Validation with qPCR

**Figure 3:**
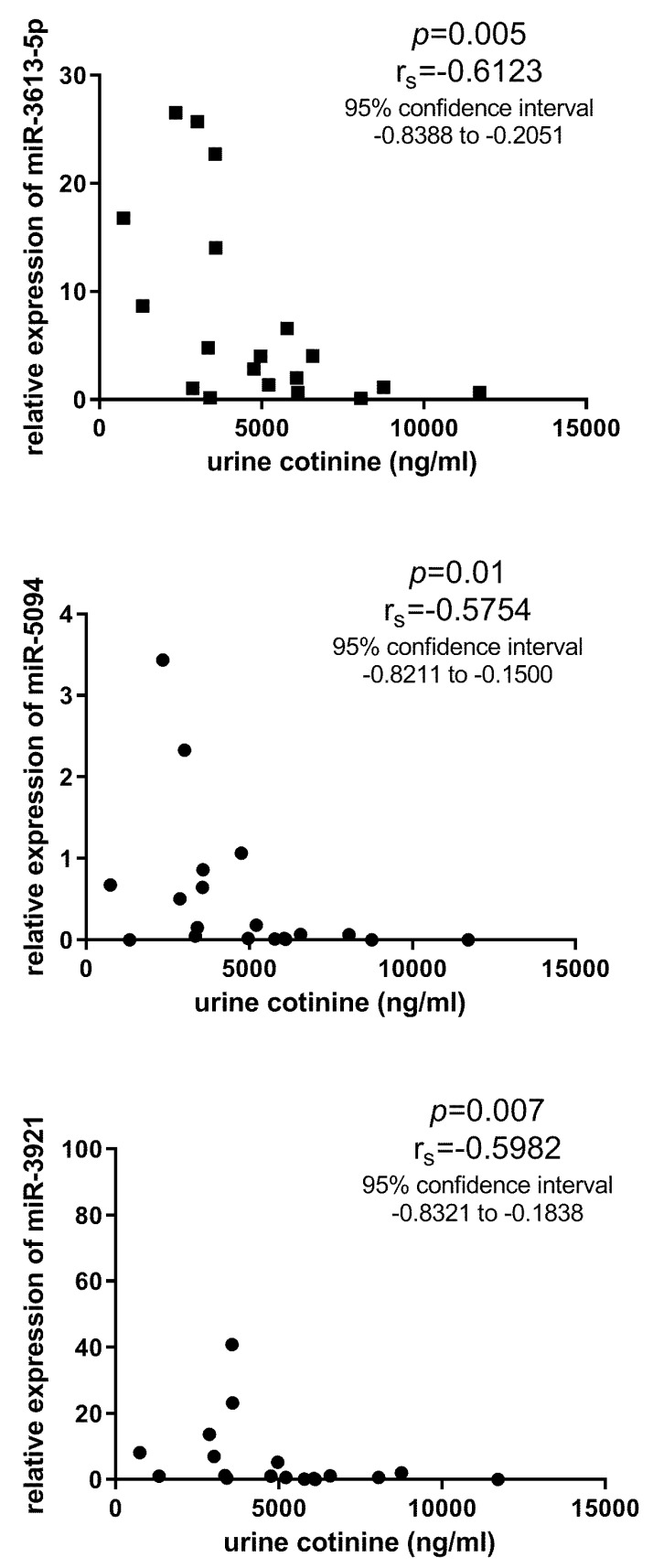
The correlations between three urine miRNAs and cotinine levels in a validation analysis.

**Figure 4:**
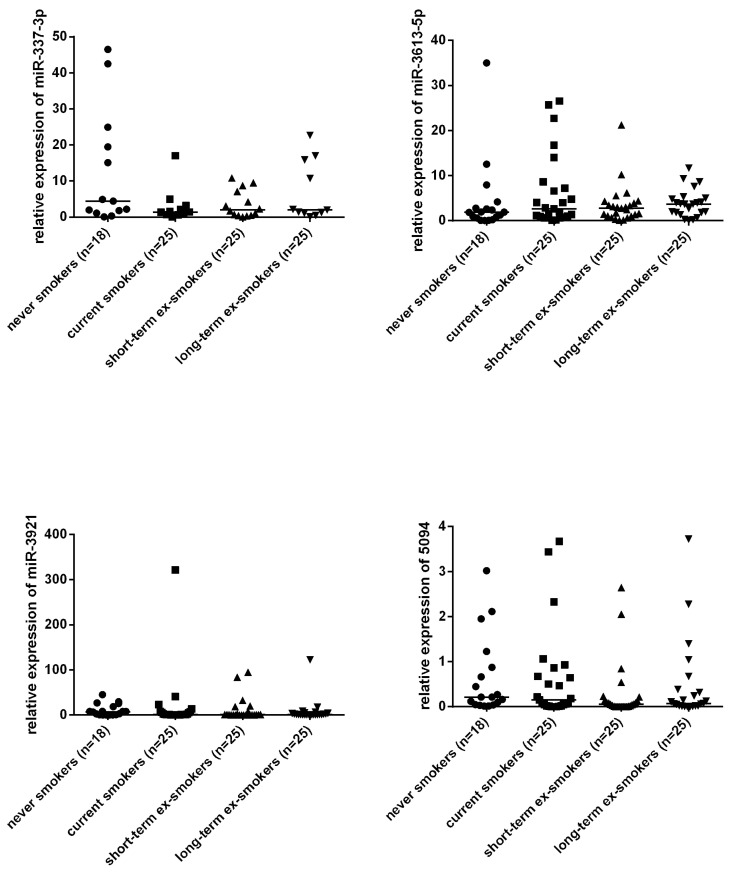
The comparison of four urine miRNAs among never smokers, current smokers, and ex-smokers in a validation analysis.

For validation, four miRNAs (hsa-miR-3613-5p, hsa-miR-3921, hsa-miR-5094, and hsa-miR-337-3p) were selected and assessed by qPCR in a larger number of individuals. Three miRNAs (hsa-miR-3613-5p, hsa-miR-3921, and hsa-miR-5094) correlated negatively with the cotinine levels in the current smokers (Figure 3). However, none of the four miRNAs was confirmed to be dysregulated between current smokers and never smokers (Figure 4). Four miRNAs were also affected by neither short-term nor long-term abstinence.

## 4. Discussion

This paper is the first investigation of urine miRNAs in tobacco smokers. The previous papers on tobacco smoking focused primarily on intracellular miRNAs in the lung or circulation [36,37,38,39]. In particular, the paper by Wang et al. (2015) reported 34 of 1100 miRNAs to be changed in the small airway epithelium obtained from smokers. Due to smoking cessation, 22 of the 34 smoking responsive miRNAs returned to the expression level of healthy non-smokers. The reversible changes confirm the direct effect of tobacco smoking on miRNA expression in the lung. Willinger et al. screened whole blood-derived miRNAs from 5023 Framingham Heart Study participants. Of 283 miRNAs, they showed six smoking-associated miRNAs that may serve as mediators of smoking-induced inflammation and this process induces organ damage [40]. Another paper by Su et al. (2016) used a genome-wide miRNA expression profile of peripheral blood mononuclear cells and reported 25 microRNAs in humans to be differentially expressed before and after smoke reduction [37]. These papers show that miRNA alternations associated with tobacco smoking are also present in the peripheral blood samples. It is in line with the paper by Izzotti et al. (2018) who used a mice model exposed to mainstream cigarette smoke. They investigated and compared 10 organs and three body fluids. The lung was the main target affected by smoke and then this was followed by skeletal muscle, liver, blood serum, kidney, spleen, stomach, heart, bronchoalveolar lavage fluid, urine, urinary bladder, and colon. Although only a small number of urinal miRNA were affected by cigarette smoke, they were clearly detectable as early as four weeks after cigarette smoke exposure, whereas the lung and blood serum were mainly detectable after eight weeks [41].

Our screening data showed a few urinal miRNAs to differ in current/ex-smokers in comparison to never smokers. Then, we enrolled the higher number of individuals in validation analysis than in our screening. Three urinal miRNAs were correlated with a metabolite of nicotine (urine cotinine) among the current smokers. However, they did not differ in current/ex-smokers in comparison to never smokers. The paired data obtained by repeated sampling of the same subject is a great advantage of this study. Each current smoker was sampled repeatedly (before and twice after quitting) to reduce the side effect of possible variables that can be hardly controlled (e.g., diet habits). Neither short-term nor long-term abstinence from tobacco smoking affected the urinal miRNAs. Furthermore, the repeated samples often cluster together in our screening analysis, e.g., the patients with numbers 14, 21, and 31. It supports the hypothesis that a patient’s samples are rather similar to each other and fewer similarity is shared among the distinct individuals despite the same smoking status. Our data, therefore, suggest that tobacco smoking has no effect or only a small effect on urinal miRNAs. Besides, our observation was not changed in the sub-analysis restricted for heavy smokers with ≥20 cigarettes per day (data not shown). The comparison between heavy smokers and never smokers is more likely to show some effect of tobacco smoke if any effect is present at all.

In the previous study by Lv et al., urine miR-29 and miR-200 were reported to robustly distinguish the patients with chronic kidney disease with high diagnostic accuracy [21]. Another study reported urinary miRNA-27b-3p and miRNA-1228-3p to correlate with the progression of kidney fibrosis in diabetic nephropathy [22]. Urine miRNAs were also suggested to serve as new biomarkers for diagnosis and recurrence prediction of bladder cancer [23,24,25,26,27,29,30]. All let-7 miRNAs are up-regulated in urine samples obtained from renal cell carcinoma [31]. Regarding pulmonary diseases, miR-337 has been associated with non-small cell lung cancer [42,43,44].

The smoking status is not stated or matched between the patients and controls in these studies. Pospisilova et al. tried to interpret their urinal data with regard to the smoking behaviour of examined subjects, but, after a critical revision of the group sizes (non-smokers, ex-smokers, and smokers), they realized that their sets are not suitable for this type of analysis due to their limited sizes and clinical heterogeneity [30].

In this case, we show that tobacco smoking does not change the urinal miRNA profile and, thus, smoking history can be omitted in future clinical studies on urine miRNAs. Based on previous studies [45,46,47], however, we can speculate on the concerned effect of tobacco smoking and age, sex, comorbidity, and others. We did not observe any of the assessed miRNAs to be dysregulated, according to sex or age. However, our study did not primarily focus on these characteristics. Regarding other studies, aging was reported to be strongly associated with circulating miRNA expression [48]. Furthermore, Turco et al. reported an increasing age to correlate with a decreasing number of renal exosome populations in the urine [49]. The exosome miRNAs that originate from the urinary tract account for the prevailing amount of urine miRNAs [50,51]. To our best knowledge, no investigation exists on the relationship between urine miRNAs and aging in clinical studies. Furthermore, the paper by M Bradicich et al. reviews 14 papers on biomarkers of smoking, e.g., exhaled CO and urine cotinine. They reported different cut-offs in healthy controls and patients with asthma and COPD [45]. The combined effect should be, therefore, the subject of other investigations before we decide definitely to exclude smoking history as a confounding factor from a future clinical investigation looking for urinal biomarkers among miRNAs. Regarding other possible confounders that could skew our observation, any individuals with urogenital diseases were excluded based on routine urine investigation and self-administered questionnaires. However, the particular values of renal urine parameters (e.g., proteinuria or glomerular filtration rate) are not available now and their absence is one of our limitations in this paper. Furthermore, our pilot data should be confirmed in a larger number of subjects who should provide at least a pair of samples before and after smoking cessation. If future studies confirm our observation, it should be mentioned that no effect or a small effect on urine miRNAs is not in contrast with the well-established benefits of smoking cessation [32,52,53].

## 5. Conclusions

In conclusion, our pilot data showed that urine miRNAs are difficult to change after smoking cessation and any difference is unlikely between current smokers and never smokers in healthy individuals. Other studies should, however, take into consideration the possibility that tobacco smoking can have a small effect on its relevance under specific conditions. We also believe that a longitudinal study design with repeated sampling provides the most reliable data and should be prioritized in future studies.

## Figures and Tables

**Table 1:** (**A**) Characteristics of the never smokers and current/ex-smokers who provided 2–3 repeated samples for screening analysis. Legend: *; numbers of smoked cigarettes per day X number of years smoked/20, CO, carbon monoxide, ppm, parts per million. (**B**) Characteristics of the never smokers and current/ex-smokers who provided three repeated samples for a validation. Legend: *, number of smoked cigarettes per day X number of years smoked/20, CO, carbon monoxide, ppm, parts per million.

**Analysed Group (Total Number of Samples, n)**	**Age (Years) Mean (Min.-Max.)**	**Sex (F:M)**	Pack-Year Mean * (Min.-Max.)	**Urine Cotinine (ng/mL) Mean (SD, Min.-Max.)**	**Exhaled CO (ppm) Mean (SD, Min.-Max.)**	**Urine Creatinine (mmol/l) (SD, Min.-Max.]**
Never smokers (6)	54 (39–83)	3:3	0	<10	0	10.81 (4.88, 2.34–15.66)
Current smokers (10)	48 (27–73)	2:8	29 (7–83)	6140 (3184, 1325–11,712)	12.4 (7.183, 1.000–25.000)	12.86 (7.50, 2.69–29.89)
Short-term successful ex-smokers (10)	48 (27–73)	2:8	29 (7–83)	684 (1346, 10–4359)	0.400 (1.265, 0.000–4.000)	11.96 (7.99, 2.22–25.53)
Long-term successful ex-smokers (7)	46 (27–67)	2:5	33 (7–83)	20 (16, 10–54)	0.286 (0.488, 0.000–1.000)	12.88 (5.11, 3.48–18.65)
**Analysed Group (Total Number of Samples, n)**	**Age (Years) Mean (Min.-Max.)**	**Sex (F:M)**	Pack-Year * Mean (Min.-Max.)	**Urine Cotinine (ng/mL) Mean (SD, Min.-Max.)**	**Exhaled CO (ppm) Mean (SD, Min.-Max.)**	**Urine Creatinine (mmol/l) (SD, Min.-Max.)**
Never smokers (18)	50 (38–83)	9:9	0	<10	0	10.81 (4.88, 2.34–15.66)
Current smokers (25)	47 (25–69)	10:15	35 (5–170)	4851 (2689, 736–11,712)	8.857 (6.139, 1.000–25.000)	10.71 (7.95, 2.05–29.89)
Short-term successful ex-smokers (25)	47 (25–69)	10:15	35 (5–170)	546 (875, 12–3285)	0.286 (0.810, 0.000–4.000)	9.69 (5.74, 2.22–22.54)
Long-term successful ex-smokers (25)	47 (25–69)	10:15	35 (5–170)	24 (15, 14–54)	0.107 (0.315, 0.000–1.000)	12.87 (4.36, 3.48– 8.65)

**Table 2:** The most dysregulated urine miRNAs after correction for multiple testing (*pc* < 0.05) between current/short-term/long-term ex-smokers versus never smokers in the base-line analysis.

Vs. Never Smokers	Mature miRNA	logFC	*p* Value	adj. P. Val
current smokers	hsa-miR-3613-5p	−4.86	3.30e-10	8.51e-07
	hsa-miR-3921	−4.14	5.43e-07	6.99e-04
	hsa-miR-5094	−1.09	1.12e-06	9.65e-04
	hsa-miR-337-3p	−2.38	2.84e-06	1.83e-03
	hsa-miR-3620-5p	1.64	7.19e-05	3.71e-02
short-term ex-smokers	hsa-miR-3613-5p	−5.77	5.58e-12	1.44e-08
	hsa-miR-337-3p	−2.70	3.11e-07	3.04e-04
	hsa-miR-3921	−4.24	3.54e-07	3.04e-04
	hsa-miR-5094	−1.13	6.26e-07	4.04e-04
	hsa-miR-7159-5p	−0.44	1.37e-05	7.05e-03
	hsa-miR-1298-3p	−1.70	6.77e-05	2.91e-02
	hsa-miR-5004-5p	−0.55	1.37e-04	0.05
	hsa-miR-6501-3p	−0.40	1.64e-04	0.05
	hsa-miR-3620-5p	1.51	2.05e-04	0.06
long-term ex-smokers	hsa-miR-3613-5p	−5.33	2.23e-10	5.75e-07
	hsa-miR-5094	−0.90	6.45e-05	0.08

## Supplementary Materials

The following are available online at https://www.mdpi.com/2075-1729/10/9/191/s1. Figure S1: The comparison of current smokers versus never smokers in base-line analysis. Legend: The urine miRNAs that differed between current smokers versus never smokers in base-line analysis (*p* < 0.005). The number following the decimal point refers to the smoking status as following: 0 (pink), never smoker and 1 (yellow), current smokers. Figure S2: The comparison of short-term ex-smokers versus never smokers in a base-line analysis. Legend: The urine miRNAs that differed between short-term ex-smokers versus never smokers in a base-line analysis (*p* < 0.005). The number following the decimal point refers to the smoking status as following: 0 (pink), never smoker, and 2 (light blue), short-term ex-smokers. Figure S3: The comparison of long-term ex-smokers versus never smokers in a base-line analysis. Legend: The urine miRNAs that differed between long-term ex-smokers versus never smokers in a base-line analysis (*p* < 0.005). The number following the decimal point refers to the smoking status as following: 0 (pink), never smoker, and 3 (dark blue), long term ex-smokers. Figure S4: The comparison of current smokers versus short-term ex-smokers in a longitudinal analysis focused on the short-term effect of smoking cessation. Legend: The number following the decimal point refers to the smoking status as following: 1 (yellow), current smokers, and 2 (light blue), short-term ex-smokers. Figure S5: The comparison of current smokers versus long-term ex-smokers in the longitudinal analysis focused on the long-term effect of smoking cessation. Legend: The number following the decimal point refers to the smoking status as following: 1 (yellow), current smokers, and 3 (dark blue), long term ex-smokers. Table S1: miRNA expression values for screening analysis.
