# Supplementary figures and images for: The Effect of Tobacco Smoking and Smoking Cessation on Urinal miRNAs in a Pilot Study
Source: Life (Basel). 2020 Sep 10;10(9):191. doi: 10.3390/life10090191 (PMC7554876; doi:10.3390/life10090191)

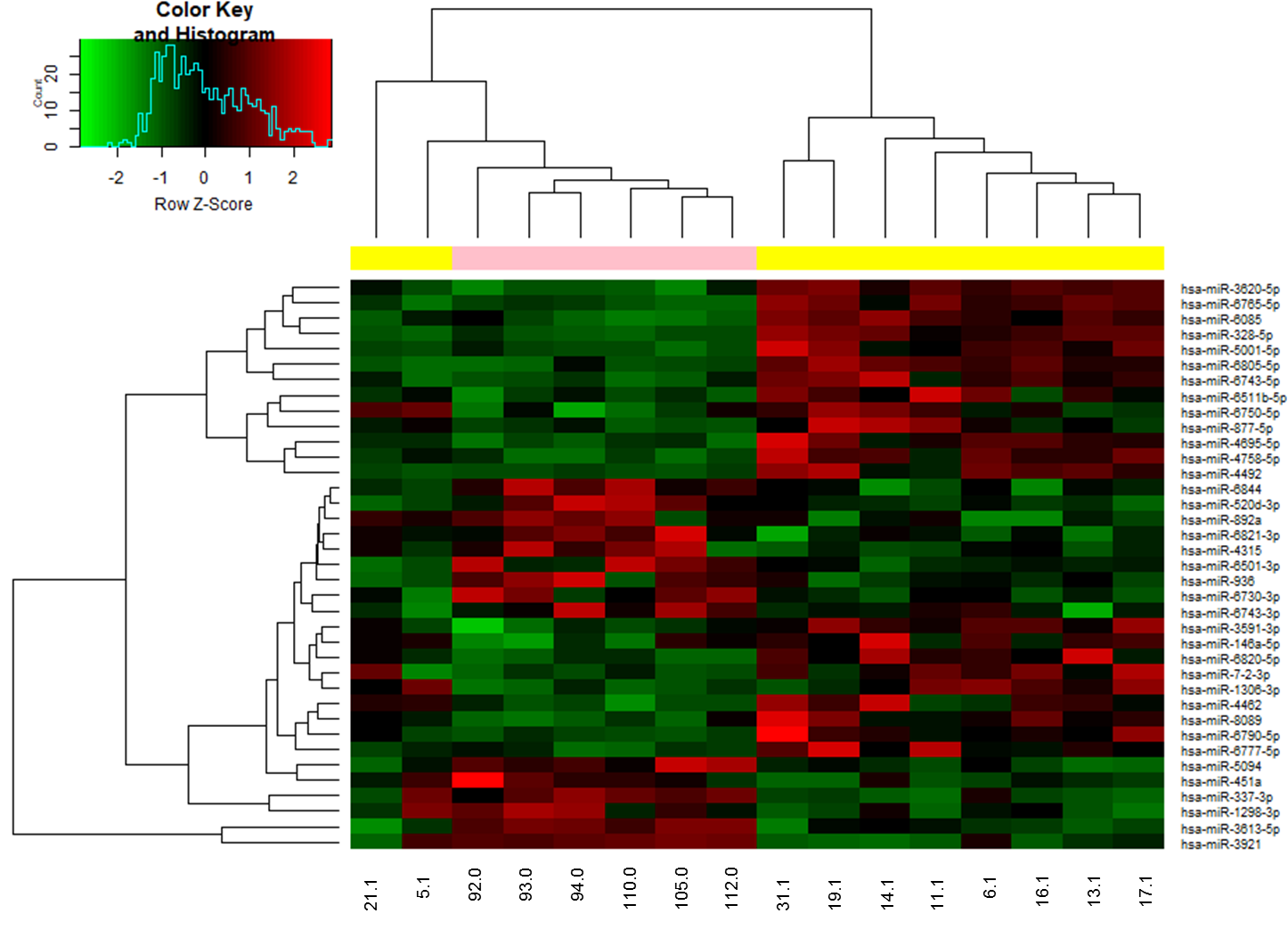

Supplement: Supplementary file 1 [file life-10-00191-s001.zip › supplement/Fig S1.tif]

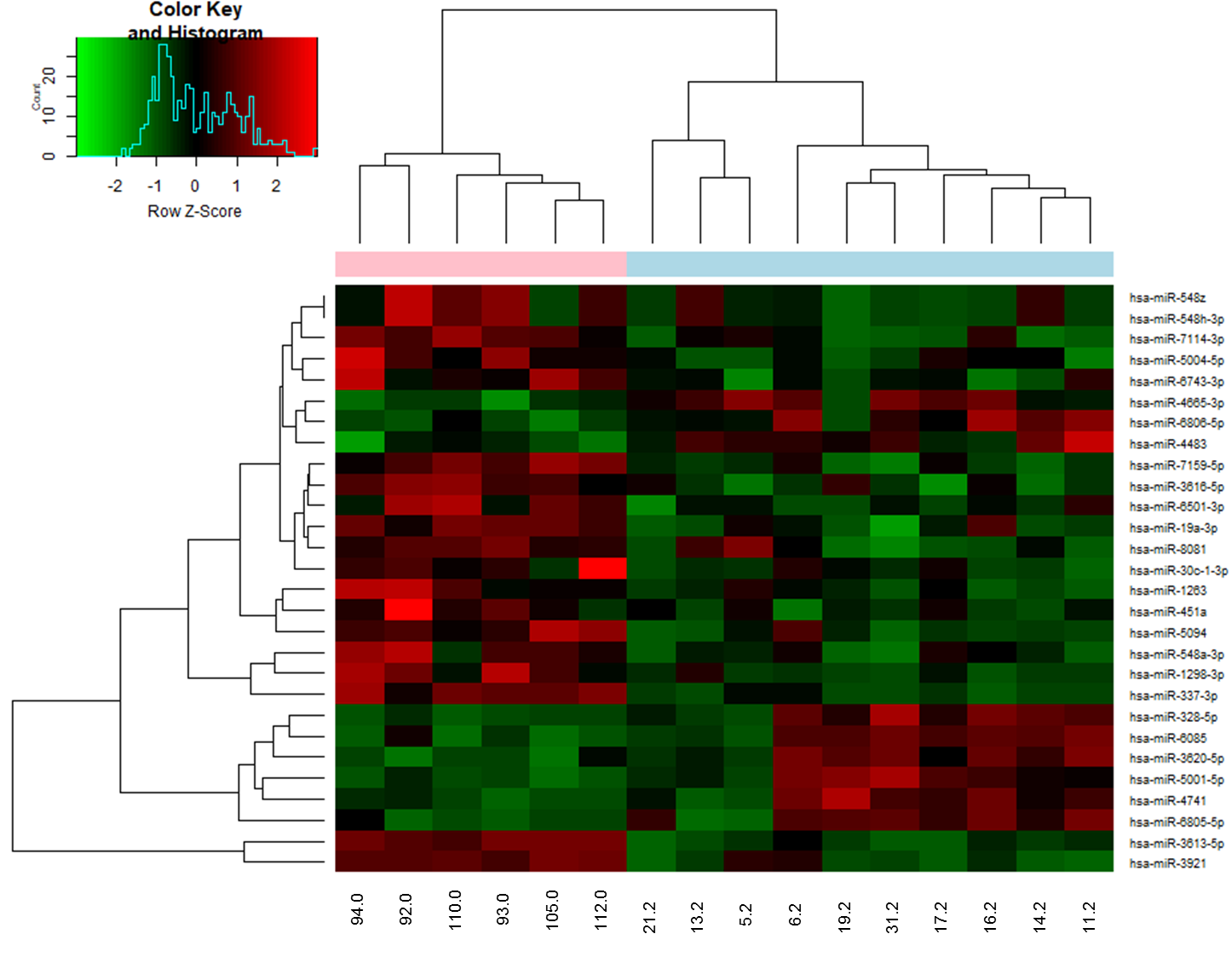

Supplement: Supplementary file 1 [file life-10-00191-s001.zip › supplement/Fig.S2.tif]

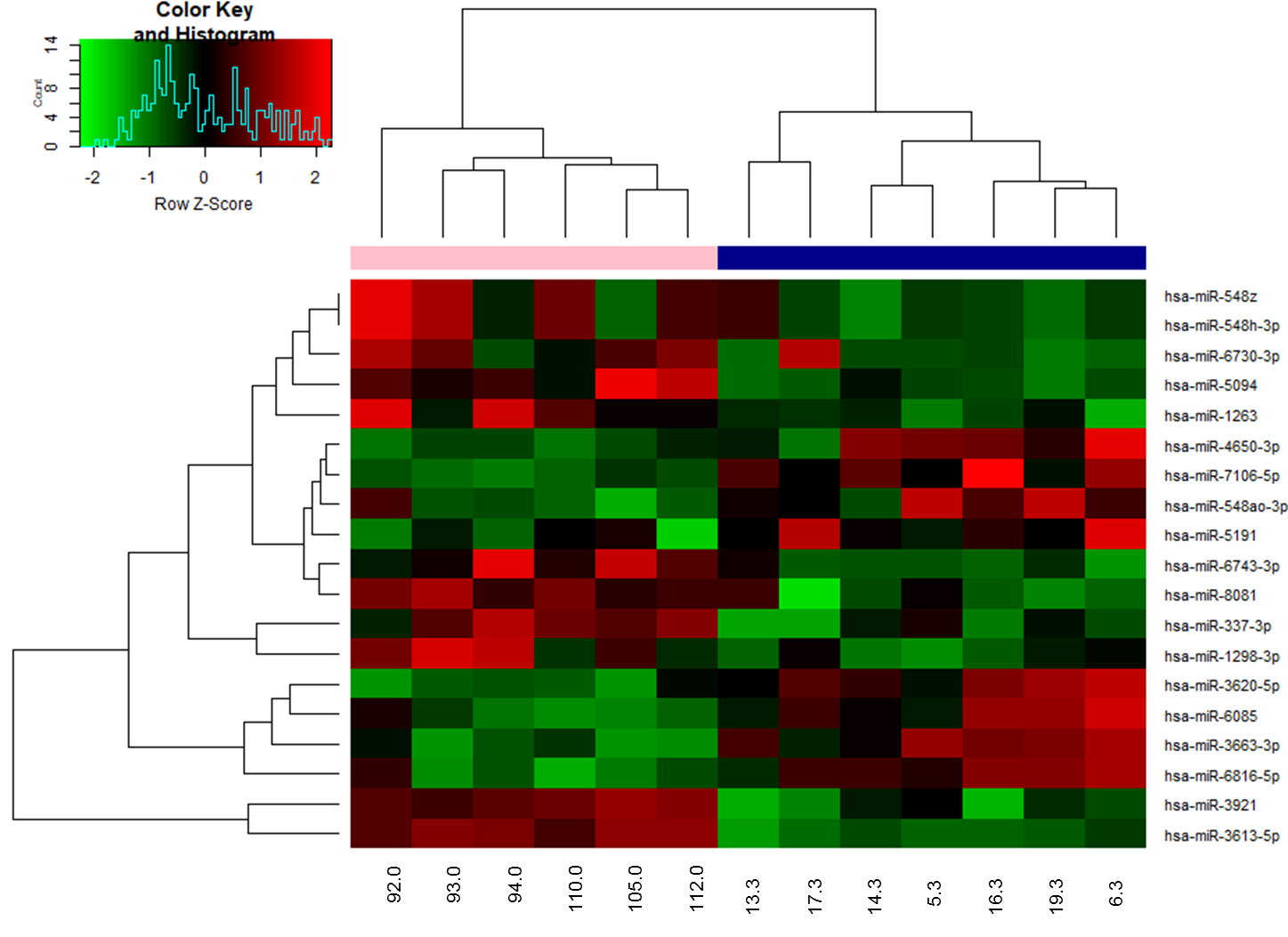

Supplement: Supplementary file 1 [file life-10-00191-s001.zip › supplement/Fig.S3.tif]

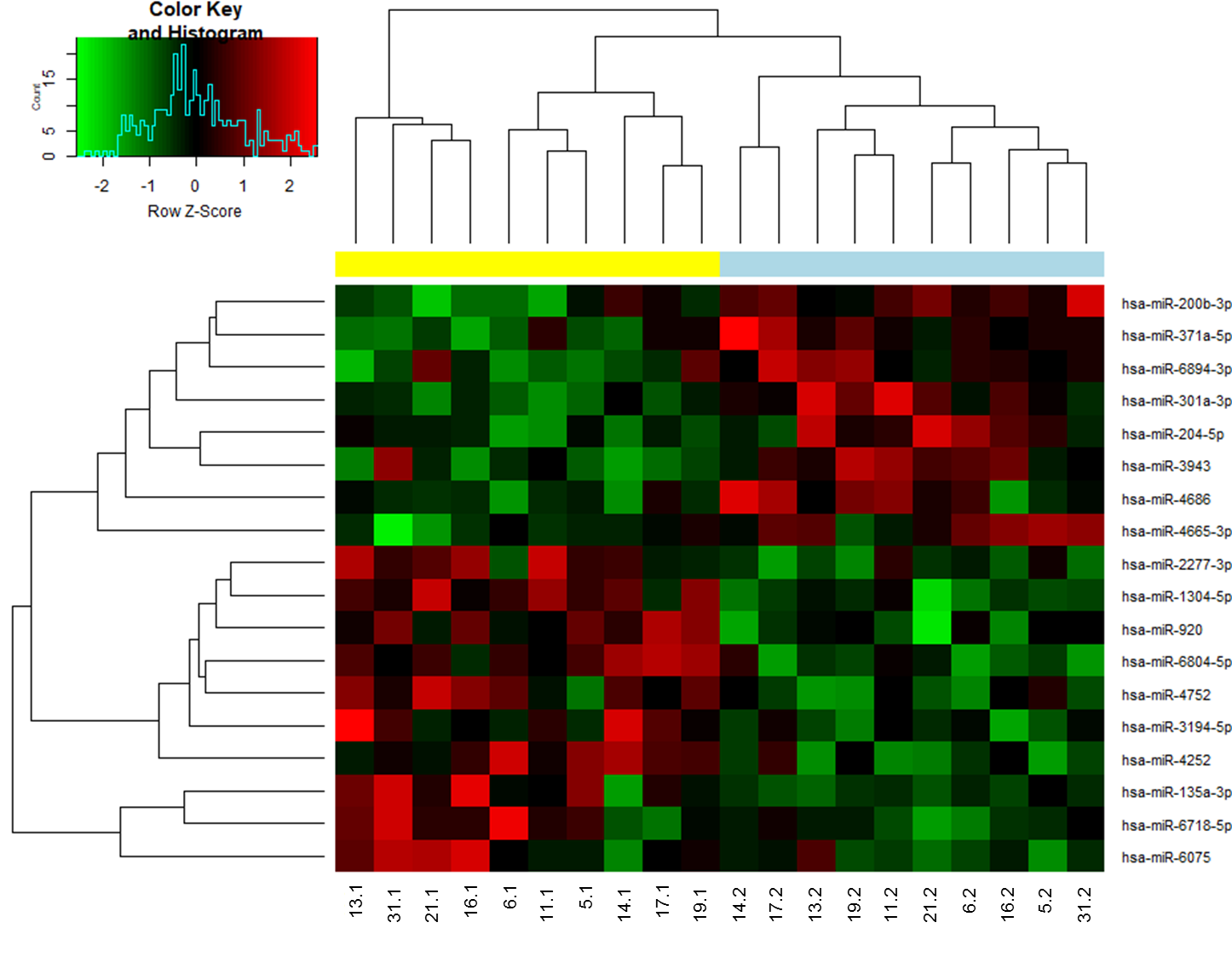

Supplement: Supplementary file 1 [file life-10-00191-s001.zip › supplement/Fig.S4.tif]

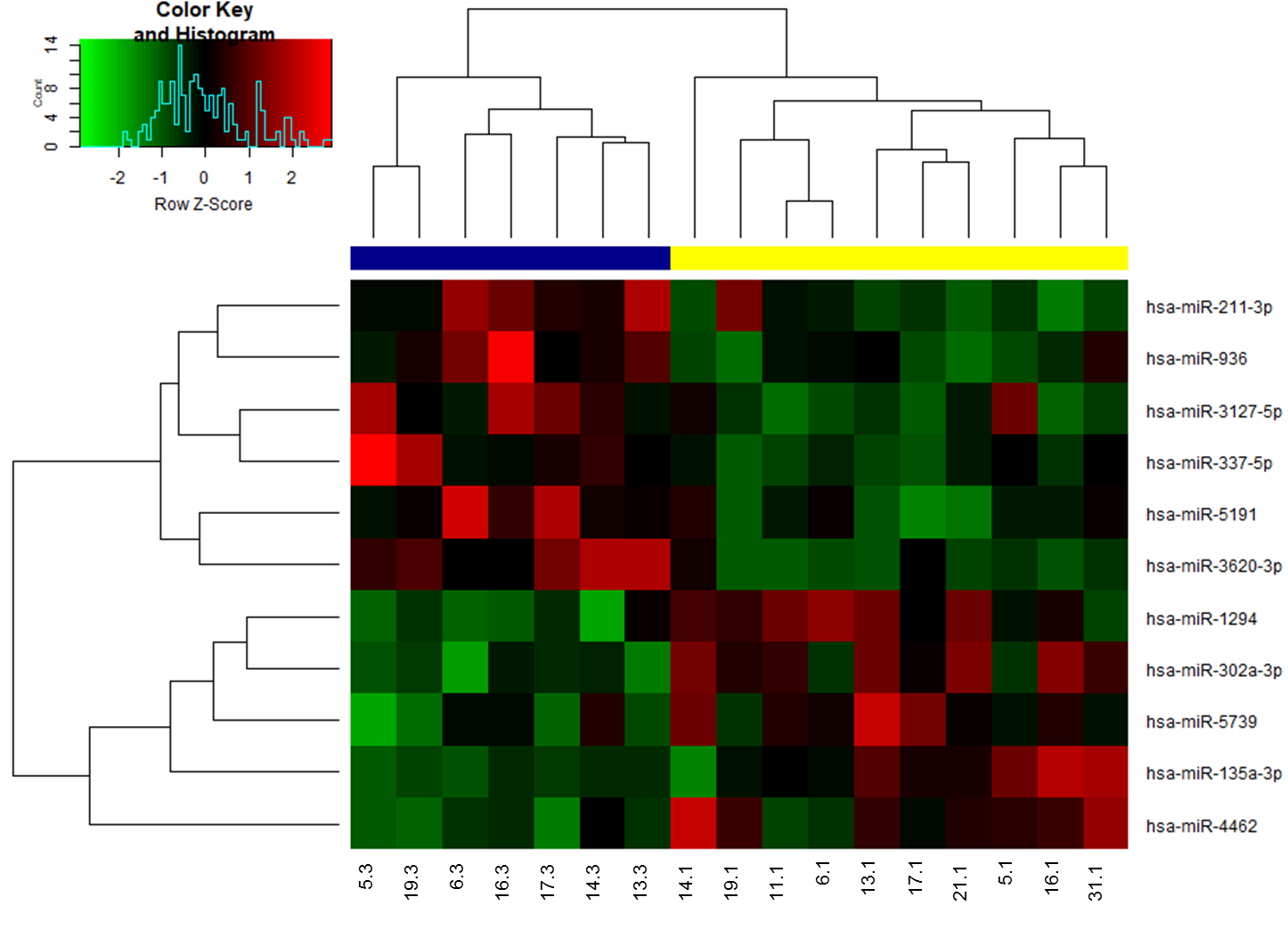

Supplement: Supplementary file 1 [file life-10-00191-s001.zip › supplement/Fig.S5.tif]
